# Within-object element ambiguity allows for a strange illusion of alternating facial expression and structure

**DOI:** 10.3389/fnhum.2022.956036

**Published:** 2022-11-07

**Authors:** Talis Bachmann

**Affiliations:** Institute of Psychology, University of Tartu, Tartu, Estonia

**Keywords:** illusion, visual perception, perceptual organization, facial expression, ambiguous figures, multistability, consciousness

## Introduction

There is abundance of objects with their perceptual attributes hierarchically organized. A tree has its trunk, branches and leaves; a car has its caroserie, doors and lights; a face has its elements arranged into a global configuration. In other words, typical objects are organized as a set of elements interrelated in certain specific ways where the elements as well as their arrangement(s) provide useful information for extracting perceptual meaning and interpretation. (Depending on the research tradition, elements may be called local features or parts whereas feature combinations be termed patterns or wholes.) The build and functioning principles of the mechanisms and algorithms dedicated to feature-, pattern- and whole-object processing have been among the basic topics of research in the neuroscience and psychology of perception (Palmer, 1999; Chalupa and Werner, 2004; Werner and Chalupa, 2014; Wagemans, 2015). In this respective research, perception of ambiguous objects has had a special status because studying alternations of subjective perceptual interpretation at the object-level processing as based on a physically invariant image allows elegant experimental disentangling of low level and higher level vision (Leopold and Logothetis, 1999; Blake and Logothetis, 2002; Long and Toppino, 2004; Sterzer et al., 2009; Kornmeier and Bach, 2012). This rests on the possibility of experimental control over low level features while higher level object interpretation varies, which helps avoid low level stimulus attributes' variablity as a confounding factor when studying the mechanisms of high level vision.

In order to conduct respective experimental studies, pertinent stimuli—i.e., specific visual images allowing alternative perceptual interpretations—have to be available for researchers. Actually, the list of such images is surprisingly long (Fisher, 1968; Bach, 1997; Long and Toppino, 2004). Among the best-known examples there are Necker cube, Schröder's stairs, Rubin's vase/faces, rabbit/duck (popularized by Jastrow), wife/mother-in-law (popularized by Boring), man/mouse (Bugelski and Alampay), and face/nude (Fisher), but the list extends to hundreds of such images. Therefore, it may seem that this list is exhaustive for experimenters with different kinds of research interests and any newly added ambiguous figure does not add much in terms of usability for specific theoretical interests. This refers especially to faces as objects, which are almost prevalent among the ambiguous stimuli. However, as will be shown further on in this paper there is still room for facial images offering novel aspects for research on perceptual bistability with physically invariant visual images. Finally, the perspectives of research offered by an ambiguous image introduced in this paper will be also discussed.

## The Merry-or-Worry illusion and its non-typical characteristics

### The stimulus image

**Figure 1:**
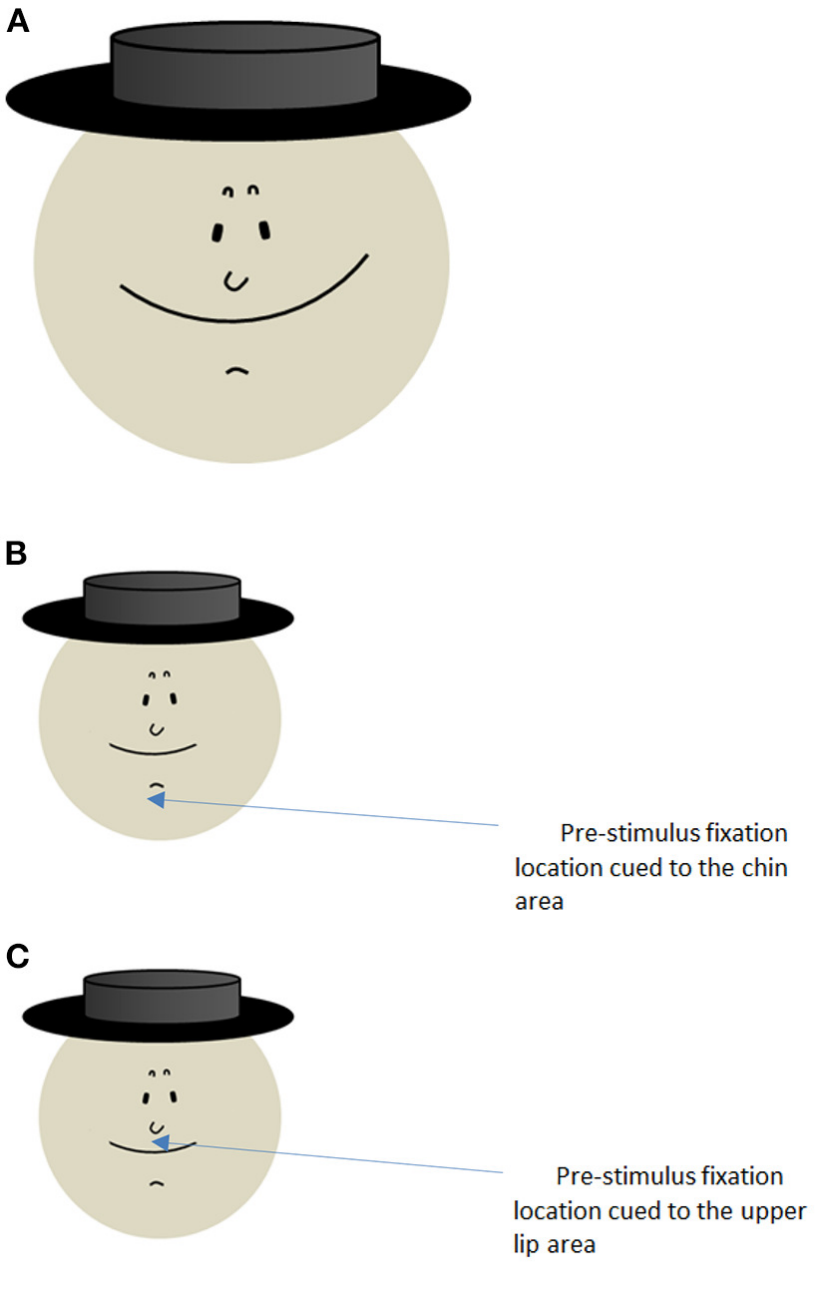
Examples of schematic facial images allowing for ambiguity in facial expression perception. **(A)** Merry-or-Worry image adapted from Bachmann (2022); **(B,C)** depict faces with subdued conspicuity of the facial element below the nose, shown in an informal study where pre-stimulus fixation location of the gaze was varied in order to direct perceptual focus.

Figure 1A depicts a drawing of a face, allowing perception of two different facial expressions by alternative interpretations of the features of an invariant image (Bachmann, 2022). Observers can experience either that the face appears as merry (a much more likely initial interpretation according to informal observations of the present author—out of the 30 students 29 said the face was merry) or as worrying or sad. To experience the illusion of change in the perceived expression, the merry-face mouth has to be reinterpreted as a mustache and the little wrinkle in the chin area as a mouth. Should the initial interpretation be a worrying face, the switch of interpretation would go *vice versa*.

### Informal further observations

When the above mentioned 30 students were asked whether they saw a mustache, all 29 who perceived the face as merry denied of seeing a mustache, but the single observer who felt the face was worrying also picked up that there was a big mustache. When the possibility of an alternative interpretation was explained to the 29 and the picture was allowed to be kept in view for as long as they wanted, majority of subjects (25) were able to switch interpretation, some with some necessary effort. Thus, the facial expression perception with this stimulus is susceptible to verbal priming and shows context dependence, consistently with top-down and constructive theories of emotion perception (Barrett, 2017; Fugate et al., 2018). In the next informal study for exploring the possible effect of within-image spatial attention two new groups of students unfamiliar with the Merry-or-Worry picture were invited to participate. The stimulus was modified in order to subdue the conspicuity of the dominant facial element because the observers in the first group did complain about the conspicuous dominance of the large smiling “mouth” (Figure 1A). (The smaller faces in Figures 1B,C depict the modified version.) Face presentation (1 s) was preceded for 4 s by a small cross-shaped pre-cue, immediately followed by the face. For one group the pre-cue directed perceptual focus on the chin area (Figure 1B), for the other group the focus was on the upper lip area (Figure 1C). With focus on chin, half of the subjects perceived the face as worrying whereas with focus on the upper lip only two subjects felt that the face was worrying—χ^2^ (1, *N* = 45) = 4.62, *p* = 0.03. Whether this effect is robust, does it depend on spatial attention or perceptual spatial resolution or does the spatial emphasis works by suggesting different virtually grouped facial elements remains to be studied in more formal and sufficiently powered research.

### Common characteristics of perceptual multistability

How this ambiguous picture compares to typical characteristics of the visual stimuli suitable for producing multistability? (The Merry-or-Worry stimulus is bistable in terms of how many *alternative interpretations* it allows and also multistable in terms of how many instances of alternative dominances can be observed. The term “multistability” can be used also to refer to multiply interpretable images with more than two interpretations available, but we leave this more complex variety aside from this paper). Huguet and Rinzel (2014) review the following such characteristics or criteria: (1) Exclusivity, meaning that conflicting visual representations are not simultaneously present in perception; (2) Inevitability, i.e., given sufficient time, switching will ultimately occur (influenced by attention and intrinsic or extrinsic biases toward one of the percepts that both can influence the dynamics of alternative interpretations); (3) Randomness, meaning that correlations between durations of successive perceptual states are absent or insignificant. The Merry-or-Worry stimulus appears to satisfy criterion (1) above according to our informal introspective observations. It also follows criterion (2) as, once reversibility of perceptual content has been discovered by the observer, sooner or later reversal(s) begin(s). For the author of this article it is possible to increase the cumulative duration of one or the other alternative by focusing voluntary attention, respectively—either at the worrisome or the merry interpretation. However, correspondence to criterion (3) has to wait for more formal assessment. In general, this illusion seems to fit with basic typical perceptual characteristics observed with ambiguous images. But are there some untypical ways of how the change between alternative variants effectively occurs with Merry-or-Worry? (We limit our discussion in this paper to the cases where ambiguity refers to perception at the level of visual objects presented by the same invariant physical depiction).

### Atypical aspects of this specific ambiguous image

Characteristically for the common ambiguous figures, when perceptual interpretation switches, the category and/or identity (individuality) of the object as perceived also changes; “packed” into the same image, there are different objects such as young and old lady, mouse and man with glasses, vase and two faces, duck and rabbit, body and face (etc.) that cannot be perceived simultaneously. In the case of our present bistable picture the object—the one face—remains the same, but its affective impression changes. This change presupposes that the meaning of the physically invariant elements (parts) of the same object subjectively changes—e.g., mustache of the worrisome looking person becomes a smiling mouth and, concomitantly, a small wrinkle in the chin area becomes a mouth (and *vice versa*). Thus, in addition to the rare case of bistability of affective expression we have also the case of change in visual virtual structure by (i) a change in elements' meanings and/or (ii) by spatial translation of the meaningful elements within the hierarchical structure *of the same object*. By default, the virtual configuration of the defining elements of the object also changes in perception. (To compare, in the rabbit/duck image the eye remains the eye, but object category and individual identity do change radically, so the change does not represent a *within-object* translation of the element, but exemplifies transfer from one object to a different object. If the meaning of the element changes—e.g., duck's beak becomes rabbit's ears—, this change is again between different objects-as-interpreted. Even though in some cases the objects' *category* remains the same between alternative interpretations like in the “wife vs. mother-in-law”, the change in individual identity of what is perceived concomitantly informs about the presence of a *different object*.) In the majority of traditional ambiguous figures the meaning of image elements changes similarly to the Merry-or-Worry picture, but the whole object identities also change, whereas the Merry-or-Worry image keeps the same object identity and reinterpretation takes place in the within-object mode. On the other hand, in such well-known examples of bistability where the perceived object remains the same, as it happens with a Necker cube, the perceived configuration of the defining features also remains the same despite the change in the subjective 3D viewpoint.

## Discussion

What kind of prospective research questions this novel multistable illusion could help to explore? Let me suggest just a few. First, in the shape of this image we seem to have a rare example where the invariance of the visual description of a common and adaptively important object, a face, is combined with variation in the meaning of its spatially localized elements. In this capacity this image as the stimulus could be instrumental in disentangling neural markers of lower level and higher level processing nodes in studies combining psychophysics and neuroscience of the correlates of conscious perception. The available neuroscience methods well suitable for this include fMRI, MEG, EEG, pupillometry. Object token and object category (and the corresponding active representational nodes in the brain) are single whereas object features exchange their receptive fields and consequently the connectivity mapping to the higher representational level also changes. This dissociation may help find neural markers of inter-level effects. Second, in many ambiguous images alternation of interpretation may be associated with virtual 3D processing (e.g., Rubin's vase/faces or Necker cube virtual rotation). The Merry-or-Worry image suggests neither a major change in 3D virtual arrangement of the perceptual viewpoint, nor a change in the figure-ground segregation in the virtual dimension of depth. Thus, this kind of stimulus can be used for experimental control of 2D vs. 3D factors in research where this attribute is of some significance. Third, as the change in perception of expression with an invariant physical stimulus speaks both to affective processing and interpersonal interaction, this stimulus could be useful for the control of visual image cues as potential confounding variables in affective and social processing research (Barrett, 2017). This is by capitalizing on the physical invariance of these cues in the stimulus image. Fourth, as the Merry-or-Worry kind of stimulus is almost unique in demonstrating multistability of affective perception (i.e., there is no need to physically vary the orientation of the stimulus elements—e.g., like in Carbon et al., 2005; Psalta et al., 2014 who used thatcherized stimuli), it can be used in studies set to compare neural correlates of processing affective and purely visual-psychophysical content.

An interesting research direction potentially capitalizing on the specifics of this kind of stimulus may be related to the “negativity bias” (e.g., Ito et al., 2017). Ito et al. (2017) presented ambiguous facial stimuli with sad and happy emotions simultaneously accessible for perception. The bias to perceive negative rather than positive emotion correlated with the activity of the bilateral pregenual anterior cingulate cortex. The Merry-or-Worry type of stimulus perception could be tried out as another index of negativity bias in relation to assessment of subjective wellbeing or such vulnerabilities as proneness to depression. For example, individuals with negativity bias may find it easier or faster to switch from merry to worry.

What are the likely mechanisms behind the Merry-or-Worry illusion? Because the adaptation and noise accumulation models (Huguet and Rinzel, 2014; Toppino and Long, 2015) are quite abstract and also quite universal they can be applied to most of the examples of ambiguous figure perception. However, as voluntary attention appears to be capable of affecting the relative dominance of alternative interpretations also with our figure and because this illusion involves unusual spatial exchange of elements' positions, the above mentioned models are insufficient. The accentuation model (Pinna et al., 2018) seems more relevant. For example, when the accentuation process is set so as to emphasize the vertically oriented grouping of eyes, nose and the wrinkle on the chin of the Merry-or-Worry, face, interpretation of worriness tends to dominate. (The accent may be made on the eyes or chin.) On the other hand, if the more horizontally oriented grouping of eyes, nose and the conspicuous arc right below nose is emphasized (e.g., by the accent on the ends of the arc or widening the element), merriness interpretation is more likely. A conceivable experiment could use biasing by creating expectation of either a face with low facial height-to-width ratio or with high value of this ratio. (The often used selective, physically produced emphasis of some parts of the face can be used as an experimental control—e.g., Leptourgos et al., 2020.) In the Gestalt tradition there are different types of cues for perceptual organization and object formation (Wagemans et al., 2012). As I noted above, the Merry-or-Worry kind of image interpretation is not so much a figure-ground phenomenon. Instead, it requires virtual perceptual grouping of selected elements into meaningful subparts of the wholistic objects according to the currently prevailing hypothesis. As this image is a physically invariant spatial arrangement of physically invariant elements within the same global object, the alternative interpretations must selectively emphasize alternative variants formed from some selected subpart of the elements just virtually. This process is steered by the currently activated meaning of the subparts of the image.

Additionally, as the top-down semantic framing by verbal instructions can influence perceptual interpretation of ambiguous figures (Mathewson, 2018), testing the susceptibility of the Merry-or-Worry illusion to this kind of manipulation might be useful for revealing its underlying higher level mechanisms. Top-down cues could be also visual of course. Substituting the hat as seen in Figure 1 by a headwear with features indicative of an old style top brass military or an artistic person could more likely activate expectation for a mustache and therefore increase prevalence of perception of worry.

Earlier research has shown that when perceptual alternation between different objects takes place (e.g., vase/faces), the winning version can be decoded already from the signals in the early visual areas (Parkkonen et al., 2008). It has to be ascertained whether also the processes specific to selection of interpretation of our *one-object* stimulus (but allowing swapping of constituent features) are distinguishable that early in the processing hierarchy. Our informal study results showing the effect of spatial perceptual focus (Figures 1B,C) suggest the possibility of early processing level contribution to the perceptual outcome. Relatedly, the temporal dynamics and time course of the Merry-or-Worry perception would also be interesting to explore. Illusory distortions of facial configuration leading to grotesque perceptual effects occur only with fast pace of alternating stimuli presentation (Tangen et al., 2011). This suggests that interpretation switch in the perception of our stimulus ought to be also fast. Experimentally flashing disambiguated merry and worrisome faces in temporally calibrated fast alternation could help find answers to this question (disambiguated versions—Bachmann, 2022).

I eagerly wait for some more formal research on the mechanisms of illusory experience carried out with a little help from the strangely ambiguous face I just introduced.

## Author contributions

The author confirms being the sole contributor of this work and has approved it for publication.

## Conflict of interest

The author declares that the research was conducted in the absence of any commercial or financial relationships that could be construed as a potential conflict of interest.

## Publisher's note

All claims expressed in this article are solely those of the authors and do not necessarily represent those of their affiliated organizations, or those of the publisher, the editors and the reviewers. Any product that may be evaluated in this article, or claim that may be made by its manufacturer, is not guaranteed or endorsed by the publisher.

## References

[B1] Bach M. (1997). Some Visual Phenomena and Optical Illusions. Available online at: http://www.michaelbach.de/ot/ (accessed October 29, 2022).

[B2] Bachmann T. (2022). Merry-or-Worry, illusion. Perception 51, 286–289. 10.1177/03010066221086080 35285746

[B3] Barrett L. F. (2017). How Emotions are Made: The Secret Life of the Brain. New York, NY: Houghton Mifflin Harcourt.

[B4] Blake R. Logothetis N. K. (2002). Visual competition. Nat. Rev. Neurosci. 3, 13–21. 10.1038/nrn701 11823801

[B5] Carbon C. C. Schweinberger S. R. Kaufmann J. M. Leder H. (2005). The Thatcher illusion seen by the brain: an event-related brain potentials study. Cogn. Brain Res. 24, 544–555. 10.1016/j.cogbrainres.2005.03.008 16099365

[B6] Chalupa L. M. Werner J. S. (2004). The Visual Neurosciences, Vols. 1 and 2. Cambridge, MA: MIT Press.

[B7] Fisher G. H. (1968). Ambiguity of form: old and new. Percept. Psychophys. 4, 189–192. 10.3758/BF03210466

[B8] Fugate J. M. B. Gendron M. Nakashima S. F. Barrett L. F. (2018). Emotion words: adding face value. Emotion 18, 693–706. 10.1037/emo0000330 28604040

[B9] Huguet G. Rinzel J. (2014). “Multistability in perception dynamics,” in Encyclopedia of Computational Neuroscience: Springer Reference, eds D. Jaeger, R. Jung (www.springerreference.com), (Berlin; Heidelberg: Springer-Verlag), 1780–1788.

[B10] Ito T. Yokokawa K. Yahata N. Isato H. Suhara T. Yamada M. (2017). Neural basis of negativity bias in the perception of ambiguous facial expression. Sci. Rep. 420, 1–9. 10.1038/s41598-017-00502-3 28341827 PMC5428736

[B11] Kornmeier J. Bach M. (2012). Ambiguous figures – what happens in the brain when perception changes but not the stimulus. Front. Hum. Neurosci. 6, 51. 10.3389/fnhum.2012.00051 22461773 PMC3309967

[B12] Leopold D. A. Logothetis N. K. (1999). Multistable phenomena: changing views in perception. Trends Cogn. Sci. 3, 254–264. 10.1016/S1364-6613(99)01332-7 10377540

[B13] Leptourgos P. Notredame C.-E. Eck M. Jardri R. Denève S. (2020). Circular inference in bistable perception. J. Vis. 20, 1–15. 10.1167/jov.20.4.12 32315404 PMC7405786

[B14] Long G. M. Toppino T. C. (2004). Enduring interest in perceptual ambiguity: alternating views of reversible figures. Psychol. Bull. 130, 748–768. 10.1037/0033-2909.130.5.748 15367079

[B15] Mathewson K. E. (2018). Duck eats rabbit: exactly which type of relational phrase can disambiguate the perception of identical side by side ambiguous figures? Perception 47, 466–469. 10.1177/0301006618756810 29402155

[B16] Palmer S. E. (1999). Vision Science: Photons to Phenomenology. Cambridge, MA: MIT Press.

[B17] Parkkonen L. Andersson J. Hämäläinen M. Hari R. (2008). Early visual brain areas reflect the percept of an ambiguous scene. Proc. Nat. Acad. Sci. 105, 20500–20504. 10.1073/pnas.0810966105 19074267 PMC2602606

[B18] Pinna B. Reeves A. Koenderink J. van Doorn A. Deiana K. (2018). A new principle of figure-ground segregation: the accentuation. Vis. Res. 143, 9–25. 10.1016/j.visres.2017.08.009 29246450

[B19] Psalta L. Young A. W. Thompson P. Andrews T. J. (2014). The thatcher illusion reveals orientation dependence in brain regions involved in processing facial expressions. Psychol. Sci. 25, 128–136. 10.1177/0956797613501521 24264941 PMC4298288

[B20] Sterzer P. Kleinschmidt A. Rees G. (2009). The neural bases of multistable perception. Trends Cogn. Sci. 13, 310–318. 10.1016/j.tics.2009.04.006 19540794

[B21] Tangen J. M. Murphy S. C. Thompson M. B. (2011). Flashed face distortion effect: grotesque faces from relative spaces. Perception 40, 628–630. 10.1068/p6968 21882726

[B22] Toppino T. C. Long G. M. (2015). Time for a change: what dominance durations reveal about adaptation effects in the perception of a bi-stable reversible figure. Attent. Perc. Psychophys. 77, 867–882. 10.3758/s13414-014-0809-x 25522830

[B23] Wagemans J. (Ed.). (2015). The Oxford Handbook of Perceptual Organization. Oxford: Oxford University Press.

[B24] Wagemans J. Feldman J. Gepshtein S. Kimchi R. Pomerantz J. R. van der Helm P. A.. (2012). A century of Gestalt psychology in visual perception: II. Conceptual and theoretical foundations. Psychol. Bull. 138, 1218–1252. 10.1037/a0029334 22845750 PMC3728284

[B25] Werner J. S. Chalupa L. M. (Eds.) (2014). The New Visual Neurosciences. Cambridge, MA: The MIT Press.

